# Synthesis of Azidohydrin from *Hura crepitans* Seed Oil: A Renewable Resource for Oleochemical Industry and Sustainable Development

**DOI:** 10.5402/2012/873046

**Published:** 2012-11-06

**Authors:** Adewale Adewuyi, Andrea Göpfert, Thomas Wolff, B. V. S. K. Rao, R. B. N. Prasad

**Affiliations:** ^1^Department of Chemical Sciences, Faculty of Natural Sciences, Redeemer's University, Ogun, Mowe 3005, Nigeria; ^2^Abteilung der Physikalischer Chemie, Technische Universität Dresden, 01062 Dresden, Germany; ^3^Centre for Lipid Research, Indian Institute of Chemical Technology, Hyderabad 500 007, India

## Abstract

The replacement of petrochemicals by oleochemical feedstocks in many industrial and domestic applications has resulted in an increase in demand for biobased products and as such recognizing and increasing the benefits of using renewable materials. In line with this, the oil extracted from the seed of *Hura crepitans* was characterized by an iodine value of 120.10 ± 0.70 g Iodine/100 g and a saponification number of 210.10 ± 0.40 mg KOH/g with the dominant fatty acid being C18:2 (52.8 ± 0.10%). The epoxidised fatty acid methyl esters prepared from the oil were used to synthesise the azidohydrin with a yield of 91.20%. The progress of the reaction was monitored and confirmed using FTIR and NMR. This showed the seed oil of *Hura crepitans* as a renewable resource that can be used to make valuable industrial and domestic products.

## 1. Introduction 

Vicinal azidoalcohols are precursors of aminoalcohols, which are well known as *β*-blockers and present in various natural products and different bioactive compounds; vicinal azidoalcohols not only constitute components of biologically active natural products but also serve as essential intermediates in the synthesis of amino sugar [1–3], carboxylic nucleosides [4], lactams [5], oxazolines, and aminoalcohols [6]. Ring-opening reactions of epoxides with nucleophiles are very useful approach in organic synthesis for the preparation of functionalized oxygenated compounds. Epoxides are versatile intermediates in organic synthesis, and their reactions with a variety of reagents such as electrophiles, nucleophiles, acids, bases, reducing agents, and some oxidizing agents are widely studied [7]. The reaction with nucleophiles such as oxygen compounds [8–10], nitrogen compounds (amine and derivatives of amines, azide, nitrate, and isocyanate), halides, and various carbon nucleophiles [11, 12] has been performed in both organic and aqueous solvents. 

Oleochemicals are chemicals derived from plant and animal fats. They are analogous to petrochemicals derived from petroleum. The formation of basic oleochemical substances like fatty acids, fatty acid methyl esters (FAMEs), fatty alcohols, fatty amines, and glycerols is by various chemical and enzymatic reactions. In the last decade, the oleochemical industry has been growing steadily due to increased global demand for more environment-friendly products. This is because of oleochemicals' attractive traits such as being derived from renewable resources, nontoxic, and readily biodegradable. Oils and fats of plant and animal origin offer possibilities of providing industries with a wealth of reaction products which will be of great value in the future. More than 90% of the hitherto published oleochemical reactions have been those occurring at the fatty acid carboxyl group while less than 10% have involved transformations of the alkyl chain [13]. There has been a lot of interest in using vegetable oils as renewable raw materials for new industrial products. It is important to develop a range of relatively facile reactions on vegetable oils in order to facilitate their use. This paper represents a step in this direction. The reaction reported appears to be a useful reaction that has many potential applications for vegetable oils, especially the lesser known underutilized ones which are potentially cheap and readily available. This trend is very positive as it has the potential for considerably extending the range of compounds that may eventually be obtainable from oils and fats thereby leading to the growth in the use of fats as renewable raw materials. 

There is much interest in the literature in the reactions of these materials to produce cost-effective derivatives [13]. Some examples are epoxidised oil [14], soybean oil methyl ester (methyl soyate) [15], maleated products [16], and derivatives of vegetable oils and soybean oil polymers [17, 18]. Preparation of azido compounds from aliphatic and aromatic epoxides obtainable from petrochemical industries has been studied [19–21]. Such compounds are good starting blocks for organic molecules to be converted into nitrogen heterocycles by decomposition or addition reactions [22, 23]. Though there have been reports on the preparation and some physical properties of azido compounds prepared from pure fatty acids and organic and polymeric substrates [24], there is however no depth of information on azido compounds prepared from the unsaturated systems in fatty acid methyl esters. Such reaction should be feasible, and it forms the objective of this study using a lesser known underutilized seed oil of *Hura crepitans* from Nigeria. This present work aimed at introducing an azido group onto the fatty acid methyl esters of *Hura crepitans* via epoxy cleavage in order to prepare vicinal amino hydroxyl methyl esters which are important in the surfactant industries. 

## 2. Materials and Methods

### 2.1. Materials

The mature seeds of *Hura crepitans *were collected from the trees grown at the garden of the University of Ibadan, Ibadan, Oyo state, Nigeria. They were identified at the Herbarium Unit, Botany Department, University of Ibadan. Formic acid (100%) and hydrogen peroxide (30%) were purchased from Merck, Darmstadt, Germany. Further chemicals and all solvents used in this study were of analytical grade and were purchased from VWR International GmbH, Darmstadt.

### 2.2. Chemical Analysis of the Oil of *Hura crepitans*

The dried seeds of *Hura crepitans *were extracted with *n*-hexane for 10 h using a soxhlet extractor [25]. The extracted oil was analyzed for its iodine, saponification, and acid values employing methods described by the Association of Official Analytical Chemists [26].

### 2.3. Fatty Acid Composition of the Oil of *Hura crepitans*

Fatty acid methyl esters of the oil were prepared by refluxing the oil at 70°C for 4 h in 2% sulphuric acid in methanol. The esters were extracted into ethyl acetate, washed free of acid, and passed over anhydrous sodium sulphate. The ethyl acetate extracts were further concentrated using a rotary evaporator. The fatty acid composition was analyzed using an Agilent 6890 N series gas chromatography equipped with FID detector on a split injector. A fused silica capillary column (DB-225, 30 m × 0.32 m i.d., J & W Scientifics, USA) was used with the injector and detector temperature maintained at 230°C and 250°C, respectively. The oven temperature was programmed at 160°C for 2 min and finally increased to 230°C at 4°C/min. The carrier gas was nitrogen at a flow rate of 1.5 mL/min. The area percentages were recorded with a standard Chemstation Data System. 

### 2.4. Methyl Esters from the Seed Oils of *Hura crepitans*

**Scheme 1:**
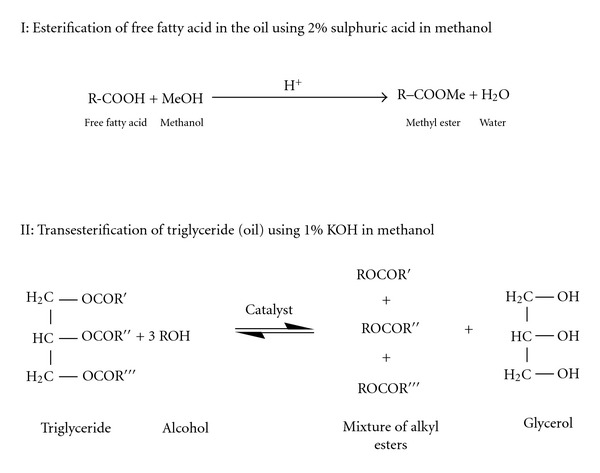
Esterification and transesterification reaction.

Methyl esters were produced from the oil of *Hura crepitans* using a two-step reaction system. The first step involved the use of 2% sulphuric acid in methanol and secondly transesterification reaction using KOH as catalyst. The oil was first esterified using 2% sulphuric acid in methanol at 70°C to convert the free fatty acid content to methyl esters. The esterification was carried out for 2 h, and the progress of the reaction was monitored using TLC to check the conversion of the free fatty acids to esters. The resultant product was extracted with ethyl acetate, washed with water until free of acid, passed over sodium sulphate, and concentrated using a rotary evaporator. The esterified oil was finally transesterified using 1% KOH in methanol at 70°C. The methyl esters formed were extracted with ethyl acetate, washed free of KOH, dried over sodium sulphate, and concentrated in a rotary evaporator. The reaction steps are shown in Scheme 1. 

### 2.5. Epoxidation of *Hura crepitans* Methyl Esters

**Scheme 2:**
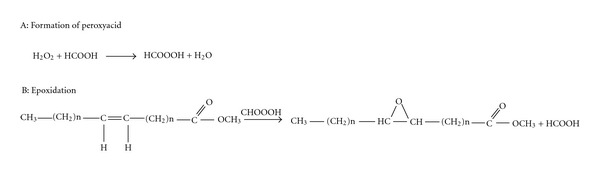
Epoxidation of fatty acid methyl esters.

The epoxidation was carried out in a 500 mL three-necked round-bottom flask equipped with a thermometer sensor and a magnetic stirrer. The methyl esters (0.0482 mol) and 100% formic acid (0.106 mol) were placed in the flask and cooled to a temperature of 15°C while stirring. Hydrogen peroxide (0.407 mol) was added dropwise with continuous stirring for about 30 min. This precaution was taken to prevent overheating of the system due to the exothermic nature of epoxidation reactions. The temperature was later raised to 70°C and maintained at this temperature for 3 h. After the formation of epoxides, the mixture was cooled to room temperature, and the epoxidised methyl esters were extracted with ethyl acetate, washed with water until free of acid, and passed over sodium sulfate. This was later concentrated using a rotary evaporator. This is shown in Scheme 2.

### 2.6. Synthesis of Azidohydrin Methyl Esters

**Scheme 3:**
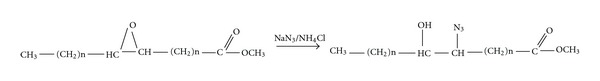
Synthesis of azidohydrin methyl esters.

The epoxidised methyl esters of *Hura crepitans* (0.042 mol) were dissolved in 50 mL of dimethylformamide in a 250 mL three-necked round-bottom flask; NH_4_Cl (0.084 mol) was added to the solution while stirring as the mixture was gradually heated and maintained at 60°C. After being heated and stirred for 10 min, NaN_3_ (0.084 mol) was gently introduced into the mixture as shown in Scheme 3. The reaction continued until all the oxirane rings had disappeared as monitored by FTIR and NMR. At the end of the reaction, the reaction mixture was allowed to cool and was later dissolved in diethyl ether in a separating funnel. The organic extract was washed with water and dried over anhydrous Na_2_SO_4_. The resulting ether layer was later concentrated using a rotary evaporator.

### 2.7. Fourier Transform Infrared (FTIR)

The FTIR spectra of oil, methyl esters, epoxidised methyl esters, and azidohydrin were recorded using AVATAR, 360 Nicolet smart Dura sample FTIR. The spectra were recorded in the range of 4000–400 cm^−1^.

### 2.8. Nuclear Magnetic Resonance (NMR) Spectroscopy

^1^HNMR and ^13^CNMR spectra of oil, methyl esters, epoxidised methyl esters, and azidohydrin were obtained using a 500 and 75 MH_Z_ Bruker Avance III Biospin AG NMR spectrophotometer in CDCl_3_ containing some amount of TMS as internal standard.

## 3. Results and Discussion

### 3.1. Extraction and Fatty Acid Composition of *Hura crepitans* Seed Oil

The yield of oil from the seed of *Hura crepitans* was 37.75 ± 0.40% iodine value and acid values were 120.10 ± 0.70 g Iodine/100 g and 4.41 ± 0.20%, respectively, while the saponification value was found to be 210.10 ± 0.40 mg KOH/g. The dominant fatty acid in the oil was C18:2 (52.8 ± 0.10%) as presented in Table 1. The iodine value sorts the oil in the range of cotton seed oil or corn oil (*Gossypium hirsutum and Zea mays*, resp.) [27, 28] while the saponification value is somewhat higher, like Tamanu oil (*Calophyllum inophyllum*) [29]. Saponification values exceeding 200 are quite rare cases, which may be important for specific industrial applications. The ease of availability, economic viability and prospect, oil yield, and high amounts of unsaturated fatty acid (82.20 ± 0.20%) were the major reasons for selecting *Hura crepitans* seed oil for the synthesis of azidohydrin; moreover, the double bonds are points at which different functional groups could be introduced into the oil.

### 3.2. Methyl Esters from *Hura crepitans* Seed Oil

**Figure 1:**
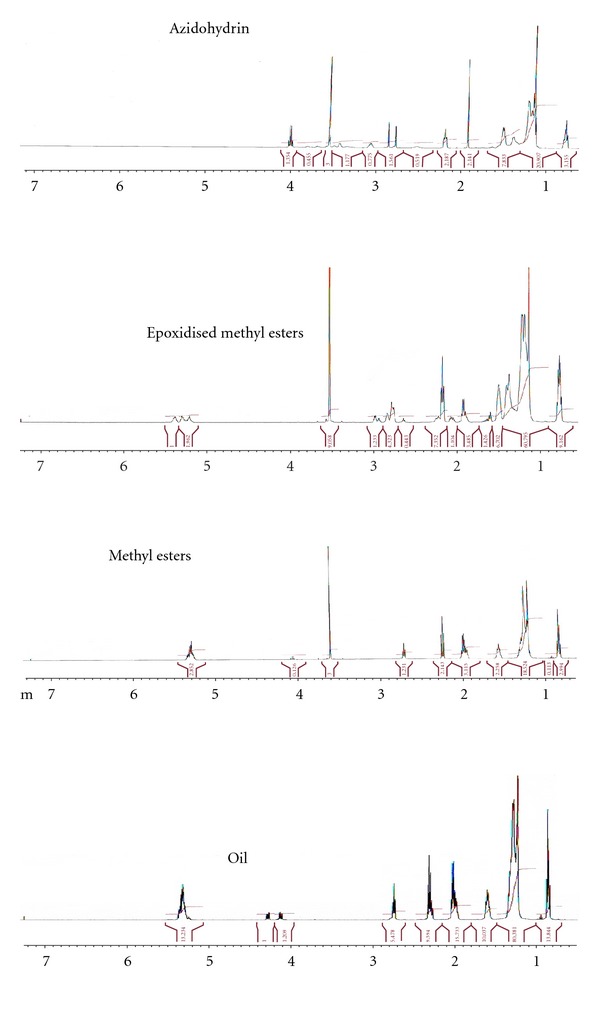
^1^HNMR spectra of the oil, methyl esters, epoxidised methyl esters, and azidohydrin of *Hura crepitans*.

The formation of the methyl esters was monitored using NMR. The ^1^HNMR spectra of the oil, methyl esters, epoxidised methyl esters, and azidohydrin are shown in Figure 1. The peaks at around 0.88, 1.25, and 1.6 ppm were attributed to the terminal methyl protons, protons of the repeating methylene units, and protons of the methylene group *β* to the carbonyl group, respectively [30, 31]. The signal at 2.0 ppm was assigned to allylic methylene protons while that at 2.7 ppm was assigned to the bisallylic methylene protons. The peak at 2.3 ppm was assigned to protons of the methylene group *α* and to the carbonyl group while peak at 5.5 ppm was considered to be from the olefinic protons, confirming the presence of unsaturation in the oil and methyl esters as indicated by the FTIR results. The signal appearing at around 4.1–4.3 ppm in the oil was assigned to the glyceridic methylene protons. An intense signal was also found at 3.7 ppm in the methyl esters which could be attributed to the protons of the methoxyl group of the esters.

**Figure 2:**
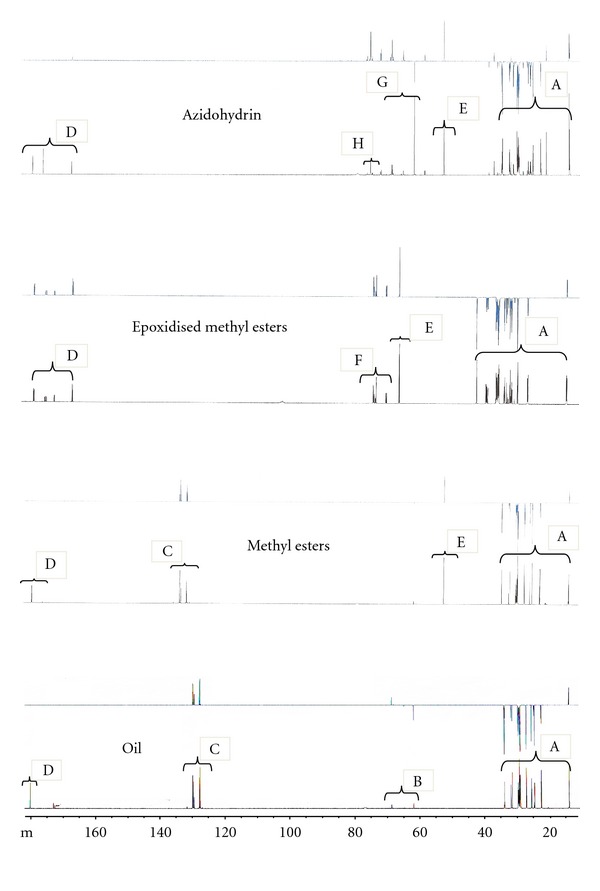
^13^CNMR spectra of the oil, methyl esters, epoxidised methyl esters and Azidohydrin of *Hura crepitans*. A: methylene and methyl carbons, B: methylene and methine carbons of glycerine moiety, C: olefinic carbon, D: carbonyl carbon, E: methoxy carbon, F: epoxy ring carbons of the methyl esters, G: carbon bearing the azide, and H: carbinol carbon.

Figure 2 presents the ^13^CNMR of the oil, methyl esters, epoxidised methyl esters, and azidohydrin. The signals at around *δ* 60.30, 62.10, and 69.20 in the ^13^CNMR spectra of the oil and methyl esters revealed the presence of glyceryl carbon atoms in the triglyceride molecules. Disappearance of the glyceryl carbon signals in the oil spectrum and appearance of new signal at *δ* 51.60 (E) in the spectrum of the methyl esters could be accounted for as being due to methoxy carbon which is indicative of the formation of the methyl esters of fatty acids supporting the result of the ^1^HNMR (3.5 ppm).

### 3.3. Epoxidation of the Methyl Esters of *Hura crepitans*

**Figure 3:**
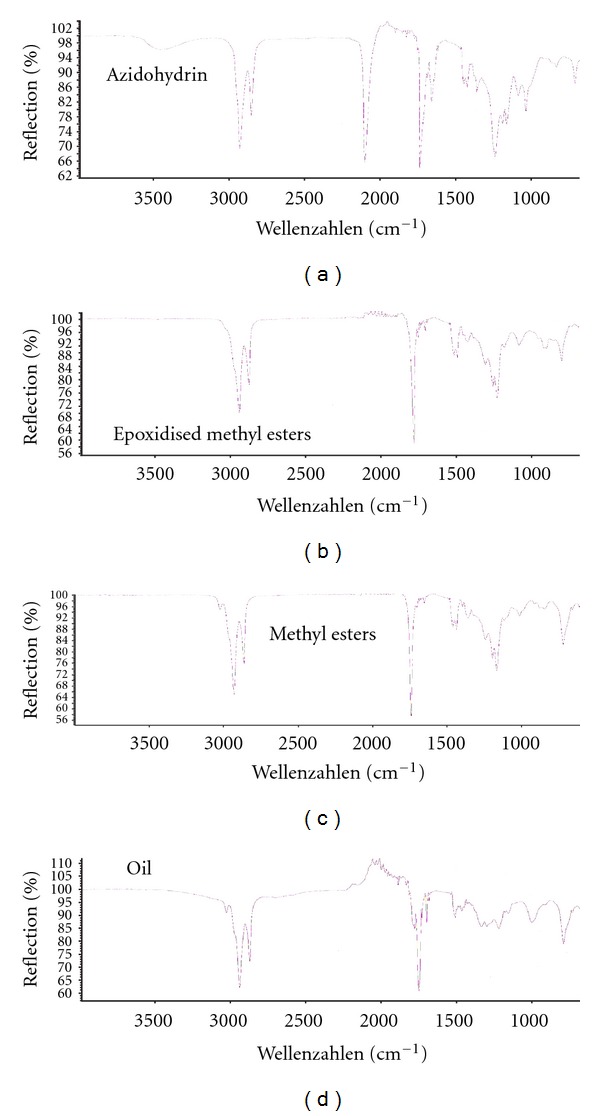
FTIR spectra of the oil, methyl esters, epoxidised methyl esters, and azidohydrin of *Hura crepitans*.

Figure 3 shows the FTIR result of the oil, methyl esters, epoxidised methyl esters, and azidohydrin. The characteristic peak at 3003.10 cm^−1^ was attributed to the C–H stretching of C=C–H in the oil and methyl esters of *Hura crepitans* indicating the presence of unsaturated functional group. Spectrum of the epoxidised methyl esters showed complete disappearance of the peak at 3003.10 cm^−1^ after 3 h. The presence of peak at 834 cm^−1^ in the epoxidised methyl esters suggested the formation of epoxides. This peak at 834 cm^−1^ was due to the symmetric in-plane deformation of the epoxy group. Also, the peak at 1250 cm^−1^ may be attributed to the symmetric ring stretching of the epoxy group. In the ^1^HNMR, the signal of the epoxy protons was found at 2.9 ppm in the epoxidised methyl esters. The ^13^CNMR revealed signal at 55–57 ppm (F) which accounted for the epoxy ring carbons of the methyl esters.

### 3.4. Synthesis of Azidohydrin Methyl Esters

The FTIR, ^1^HNMR, and ^13^C-signal analysis confirmed the conversion of the epoxidised methyl esters to azidohydrin with a yield of 91.20%. The opening of the oxirane ring of the epoxidised methyl esters gave secondary hydroxyl group and vicinal azido group with band at around 3394 cm^−1^ and 2101 cm^−1^, respectively, while the peak at 1737 cm^−1^ was considered to be the carbonyl of ester group. In the ^1^HNMR spectra, the epoxides peak at 2.9 ppm disappeared, and the azidohydrin peak appeared at around 3.1 ppm. The signal of the secondary hydroxyl functional group was found at 3.4 ppm while the hydroxyl groups formed as a result of the opening of the epoxy ring were at 2.8 and 2.7 ppm. The ^13^C spectra revealed the carbinol peak at 73.25 and 73.30 ppm and the carbon bearing the azide resonates at 66.77 and 66.83 ppm.

The azidohydrin prepared here can serve as starting material for the production of vicinal amino hydroxyl methyl esters, which is a class of compounds frequently used in the surfactant and cosmetic industries.

## 4. Conclusion 

In the present study oil was extracted from the seed of *Hura crepitans*, which was analyzed and used in the synthesis of the corresponding azidohydrin. The oil of *Hura crepitans* has an iodine value of 120.10 ± 0.70 g Iodine/100 g and a high saponification number of 210.10 ± 0.40 mg KOH/g with the dominant fatty acid being C18:2 (52.8 ± 0.10%). A feasible synthesis of the azidohydrin is presented with a yield of 91.20%. The presented plant oil adds to the known renewable resources that can be used to make valuable products, and we anticipate that the azidohydrin synthesized will find applications in the formulation of high-performance chemical products. 

## Figures and Tables

**Table 1:** Fatty acid composition (wt%) of *Hura crepitans *seed oils.

Fatty acids	*Hura crepitans *
16:0	12.20 ± 0.20
16:1	0.10 ± 0.00
18:0	5.10 ± 0.30
18:1	27.20 ± 0.20
18:2	52.80 ± 0.10
18:3	1.80 ± 0.10
20:0	0.20 ± 0.10
20:1	0.30 ± 0.10
22:0	0.30 ± 0.10
Unsaturated	82.20 ± 0.20
Saturated	17.80 ± 0.20

Values are mean ± standard deviation of triplicate determinations.
